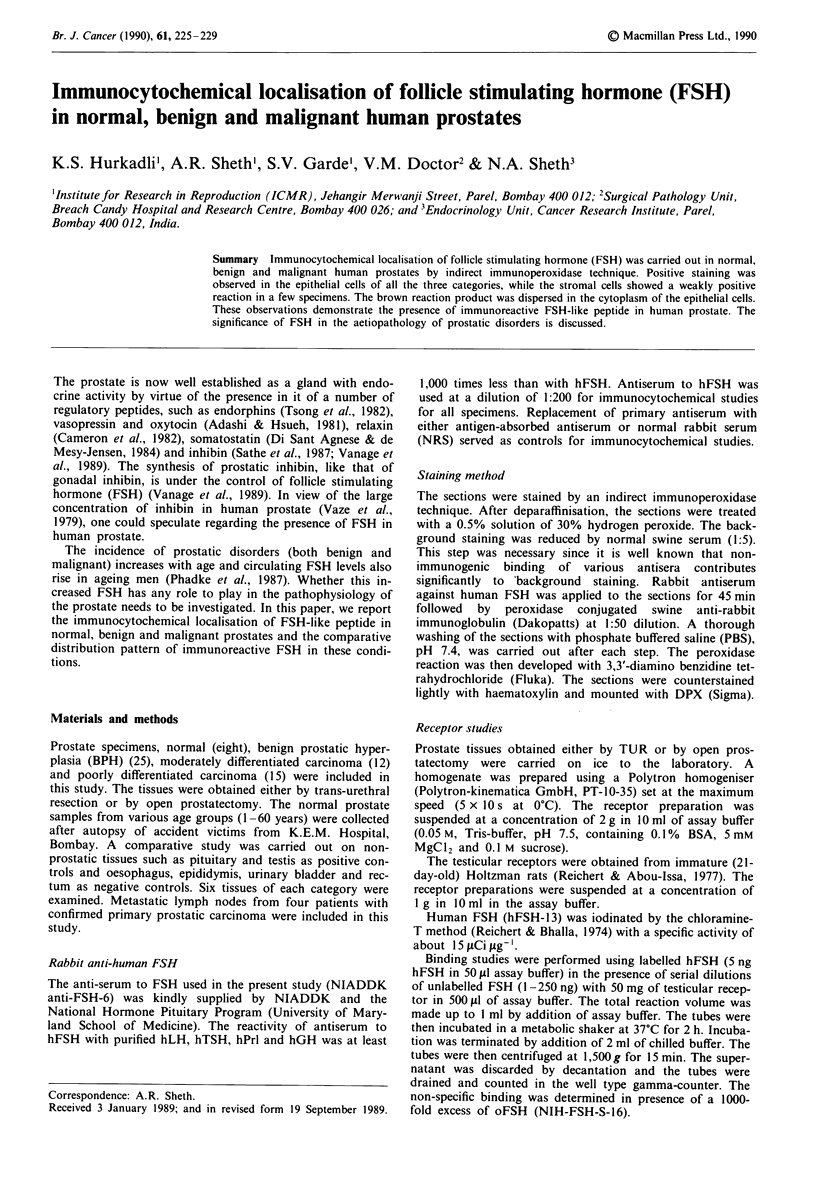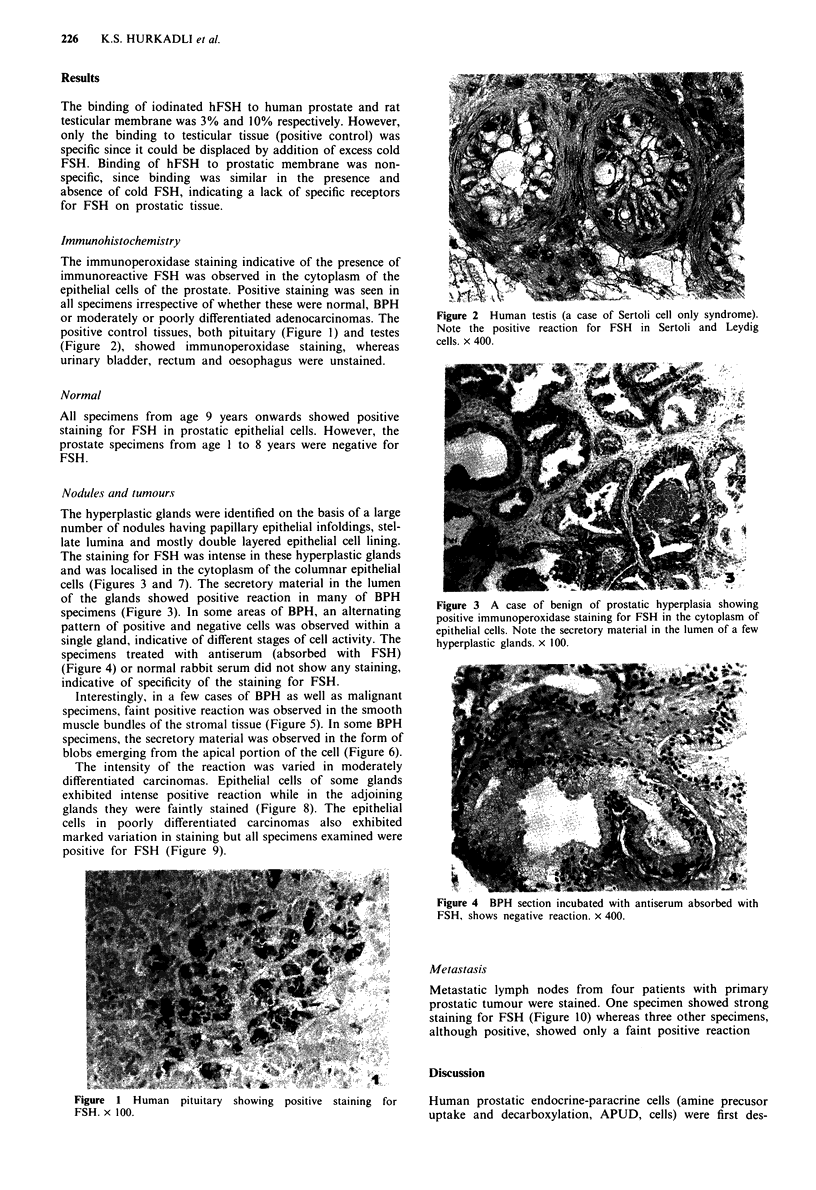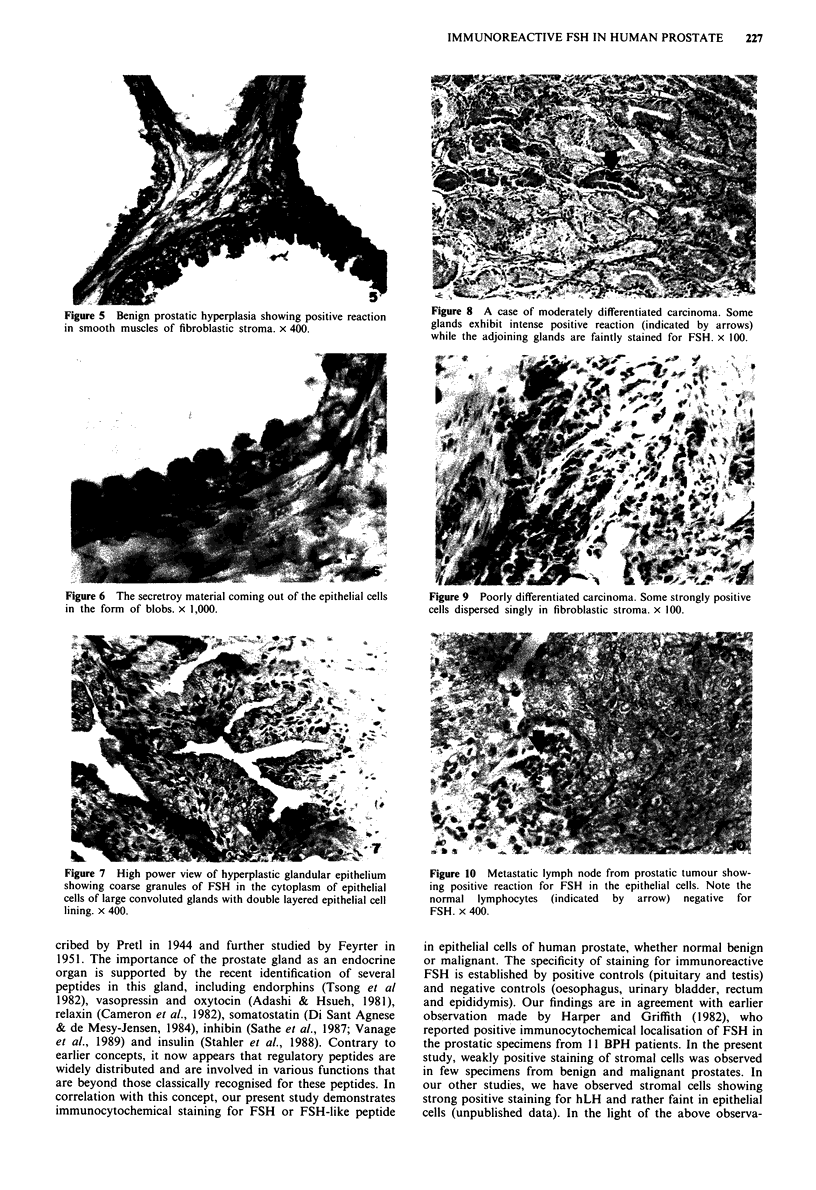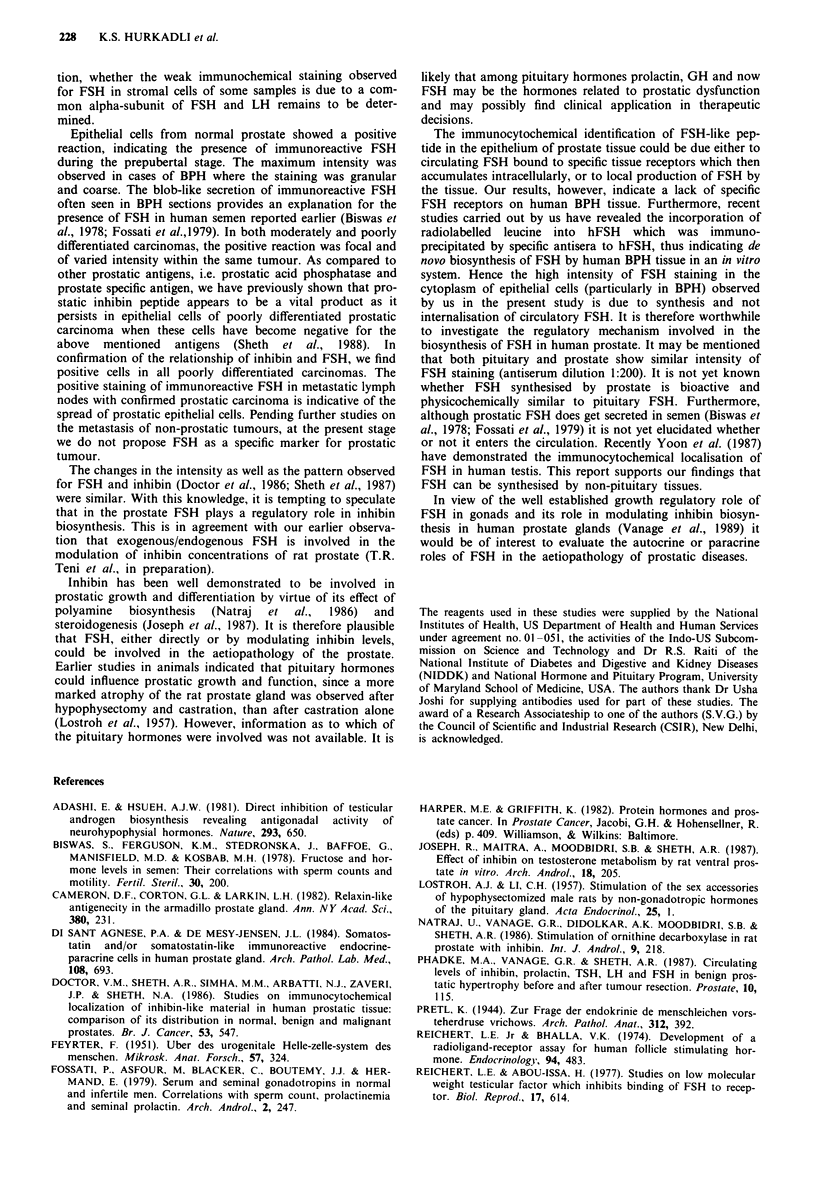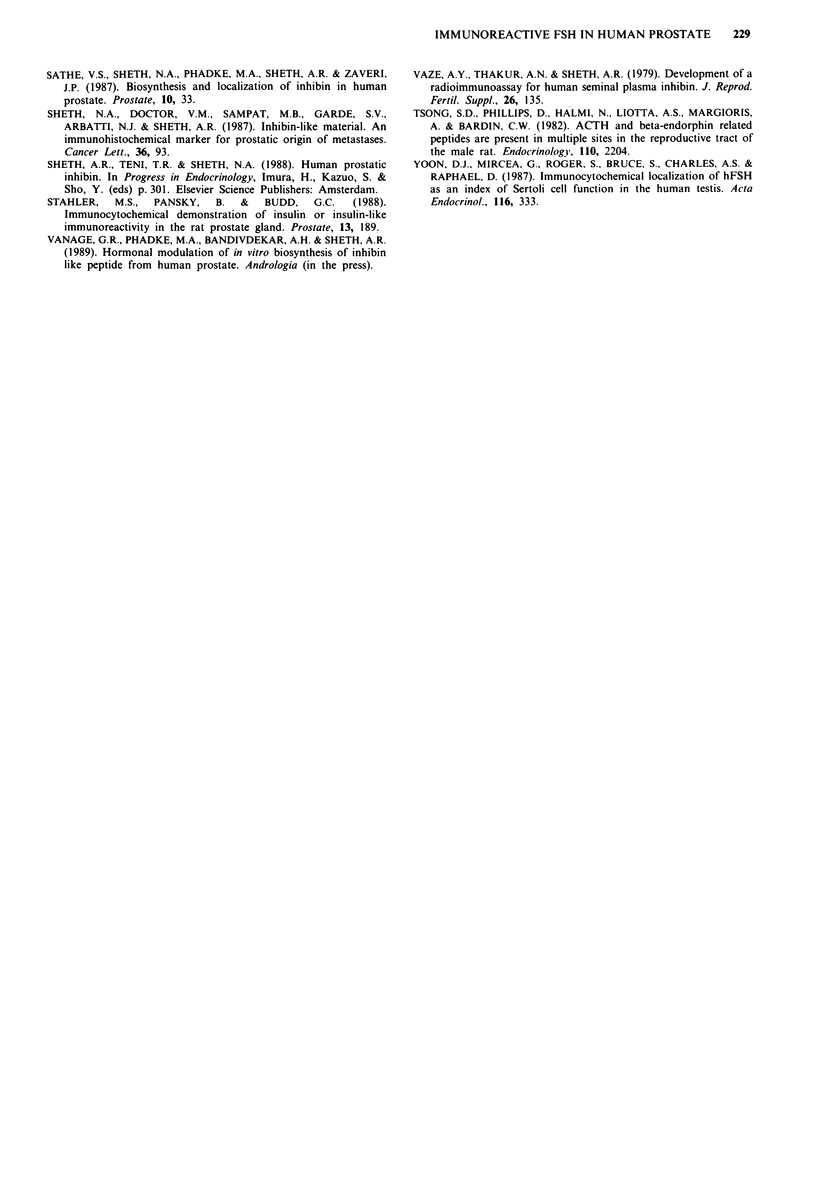# Immunocytochemical localisation of follicle stimulating hormone (FSH) in normal, benign and malignant human prostates.

**DOI:** 10.1038/bjc.1990.42

**Published:** 1990-02

**Authors:** K. S. Hurkadli, A. R. Sheth, S. V. Garde, V. M. Doctor, N. A. Sheth

**Affiliations:** Institute for Research in Reproduction ICMR, Bombay, India.

## Abstract

**Images:**


Br. J. Cancer (1990), 61, 225-229 © Macmillan Press Ltd., 1990

### Immunocytochemical localisation of follicle stimulating hormone (FSH) in normal, benign and malignant human prostates

K.S. Hurkadli[1], A.R. Sheth[1], S.V. Garde[1], V.M. Doctor[2] & N.A. Sheth[3]

[1]*Institute for Research in Reproduction (ICMR), Jehangir Merwanji Street, Parel, Bombay 400 012;* [2]*Surgical Pathology Unit, Breach Candy Hospital and Research Centre, Bombay 400 026; and* [3]*Endocrinology Unit, Cancer Research Institute, Parel, Bombay 400 012, India.*

**Summary** Immunocytochemical localisation of follicle stimulating hormone (FSH) was carried out in normal, benign and malignant human prostates by indirect immunoperoxidase technique. Positive staining was observed in the epithelial cells of all the three categories, while the stromal cells showed a weakly positive reaction in a few specimens. The brown reaction product was dispersed in the cytoplasm of the epithelial cells. These observations demonstrate the presence of immunoreactive FSH-like peptide in human prostate. The significance of FSH in the aetiopathology of prostatic disorders is discussed.

The prostate is now well established as a gland with endocrine activity by virtue of the presence in it of a number of regulatory peptides, such as endorphins (Tsong *et al.*, 1982), vasopressin and oxytocin (Adashi & Hsueh, 1981), relaxin (Cameron *et al.*, 1982), somatostatin (Di Sant Agnese & de Mesy-Jensen, 1984) and inhibin (Sathe *et al.*, 1987; Vanage *et al.*, 1989). The synthesis of prostatic inhibin, like that of gonadal inhibin, is under the control of follicle stimulating hormone (FSH) (Vanage *et al.*, 1989). In view of the large concentration of inhibin in human prostate (Vaze *et al.*, 1979), one could speculate regarding the presence of FSH in human prostate.

The incidence of prostatic disorders (both benign and malignant) increases with age and circulating FSH levels also rise in ageing men (Phadke *et al.*, 1987). Whether this increased FSH has any role to play in the pathophysiology of the prostate needs to be investigated. In this paper, we report the immunocytochemical localisation of FSH-like peptide in normal, benign and malignant prostates and the comparative distribution pattern of immunoreactive FSH in these conditions.

### Materials and methods

Prostate specimens, normal (eight), benign prostatic hyperplasia (BPH) (25), moderately differentiated carcinoma (12) and poorly differentiated carcinoma (15) were included in this study. The tissues were obtained either by trans-urethral resection or by open prostatectomy. The normal prostate samples from various age groups (1-60 years) were collected after autopsy of accident victims from K.E.M. Hospital, Bombay. A comparative study was carried out on non-prostatic tissues such as pituitary and testis as positive controls and oesophagus, epididymis, urinary bladder and rectum as negative controls. Six tissues of each category were examined. Metastatic lymph nodes from four patients with confirmed primary prostatic carcinoma were included in this study.

### *Rabbit anti-human FSH*

The anti-serum to FSH used in the present study (NIADDK anti-FSH-6) was kindly supplied by NIADDK and the National Hormone Pituitary Program (University of Maryland School of Medicine). The reactivity of antiserum to hFSH with purified hLH, hTSH, hPrl and hGH was at least 1,000 times less than with hFSH. Antiserum to hFSH was used at a dilution of 1:200 for immunocytochemical studies for all specimens. Replacement of primary antiserum with either antigen-absorbed antiserum or normal rabbit serum (NRS) served as controls for immunocytochemical studies.

### *Staining method*

The sections were stained by an indirect immunoperoxidase technique. After deparaffinisation, the sections were treated with a 0.5% solution of 30% hydrogen peroxide. The background staining was reduced by normal swine serum (1:5). This step was necessary since it is well known that non-immunogenic binding of various antisera contributes significantly to background staining. Rabbit antiserum against human FSH was applied to the sections for 45 min followed by peroxidase conjugated swine anti-rabbit immunoglobulin (Dakopatts) at 1:50 dilution. A thorough washing of the sections with phosphate buffered saline (PBS), pH 7.4, was carried out after each step. The peroxidase reaction was then developed with 3,3'-diamino benzidine tetrahydrochloride (Fluka). The sections were counterstained lightly with haematoxylin and mounted with DPX (Sigma).

### *Receptor studies*

Prostate tissues obtained either by TUR or by open prostatectomy were carried on ice to the laboratory. A homogenate was prepared using a Polytron homogeniser (Polytron-kinematica GmbH, PT-10-35) set at the maximum speed (5 × 10 s at 0°C). The receptor preparation was suspended at a concentration of 2 g in 10 ml of assay buffer (0.05 M, Tris-buffer, pH 7.5, containing 0.1% BSA, 5 mM $MgCl_2$ and 0.1 M sucrose).

The testicular receptors were obtained from immature (21-day-old) Holtzman rats (Reichert & Abou-Issa, 1977). The receptor preparations were suspended at a concentration of 1 g in 10 ml in the assay buffer.

Human FSH (hFSH-13) was iodinated by the chloramine-T method (Reichert & Bhalla, 1974) with a specific activity of about 15 $\mu Ci\ \mu g^{-1}$.

Binding studies were performed using labelled hFSH (5 ng hFSH in 50 $\mu$l assay buffer) in the presence of serial dilutions of unlabelled FSH (1-250 ng) with 50 mg of testicular receptor in 500 $\mu$l of assay buffer. The total reaction volume was made up to 1 ml by addition of assay buffer. The tubes were then incubated in a metabolic shaker at 37°C for 2 h. Incubation was terminated by addition of 2 ml of chilled buffer. The tubes were then centrifuged at 1,500 *g* for 15 min. The supernatant was discarded by decantation and the tubes were drained and counted in the well type gamma-counter. The non-specific binding was determined in presence of a 1000-fold excess of oFSH (NIH-FSH-S-16).

Correspondence: A.R. Sheth.
Received 3 January 1989; and in revised form 19 September 1989.

### Results

The binding of iodinated hFSH to human prostate and rat testicular membrane was 3% and 10% respectively. However, only the binding to testicular tissue (positive control) was specific since it could be displaced by addition of excess cold FSH. Binding of hFSH to prostatic membrane was non-specific, since binding was similar in the presence and absence of cold FSH, indicating a lack of specific receptors for FSH on prostatic tissue.

### *Immunohistochemistry*

The immunoperoxidase staining indicative of the presence of immunoreactive FSH was observed in the cytoplasm of the epithelial cells of the prostate. Positive staining was seen in all specimens irrespective of whether these were normal, BPH or moderately or poorly differentiated adenocarcinomas. The positive control tissues, both pituitary (Figure 1) and testes (Figure 2), showed immunoperoxidase staining, whereas urinary bladder, rectum and oesophagus were unstained.

### *Normal*

All specimens from age 9 years onwards showed positive staining for FSH in prostatic epithelial cells. However, the prostate specimens from age 1 to 8 years were negative for FSH.

### *Nodules and tumours*

The hyperplastic glands were identified on the basis of a large number of nodules having papillary epithelial infoldings, stellate lumina and mostly double layered epithelial cell lining. The staining for FSH was intense in these hyperplastic glands and was localised in the cytoplasm of the columnar epithelial cells (Figures 3 and 7). The secretory material in the lumen of the glands showed positive reaction in many of BPH specimens (Figure 3). In some areas of BPH, an alternating pattern of positive and negative cells was observed within a single gland, indicative of different stages of cell activity. The specimens treated with antiserum (absorbed with FSH) (Figure 4) or normal rabbit serum did not show any staining, indicative of specificity of the staining for FSH.

Interestingly, in a few cases of BPH as well as malignant specimens, faint positive reaction was observed in the smooth muscle bundles of the stromal tissue (Figure 5). In some BPH specimens, the secretory material was observed in the form of blobs emerging from the apical portion of the cell (Figure 6).

The intensity of the reaction was varied in moderately differentiated carcinomas. Epithelial cells of some glands exhibited intense positive reaction while in the adjoining glands they were faintly stained (Figure 8). The epithelial cells in poorly differentiated carcinomas also exhibited marked variation in staining but all specimens examined were positive for FSH (Figure 9).

**Figure 1** Human pituitary showing positive staining for FSH. × 100.

**Figure 2** Human testis (a case of Sertoli cell only syndrome). Note the positive reaction for FSH in Sertoli and Leydig cells. × 400.

**Figure 3** A case of benign of prostatic hyperplasia showing positive immunoperoxidase staining for FSH in the cytoplasm of epithelial cells. Note the secretory material in the lumen of a few hyperplastic glands. × 100.

**Figure 4** BPH section incubated with antiserum absorbed with FSH, shows negative reaction. × 400.

### *Metastasis*

Metastatic lymph nodes from four patients with primary prostatic tumour were stained. One specimen showed strong staining for FSH (Figure 10) whereas three other specimens, although positive, showed only a faint positive reaction

### Discussion

Human prostatic endocrine-paracrine cells (amine precusor uptake and decarboxylation, APUD, cells) were first des-

**Figure 5** Benign prostatic hyperplasia showing positive reaction in smooth muscles of fibroblastic stroma. × 400.

**Figure 6** The secretroy material coming out of the epithelial cells in the form of blobs. × 1,000.

**Figure 7** High power view of hyperplastic glandular epithelium showing coarse granules of FSH in the cytoplasm of epithelial cells of large convoluted glands with double layered epithelial cell lining. × 400.

**Figure 8** A case of moderately differentiated carcinoma. Some glands exhibit intense positive reaction (indicated by arrows) while the adjoining glands are faintly stained for FSH. × 100.

**Figure 9** Poorly differentiated carcinoma. Some strongly positive cells dispersed singly in fibroblastic stroma. × 100.

**Figure 10** Metastatic lymph node from prostatic tumour showing positive reaction for FSH in the epithelial cells. Note the normal lymphocytes (indicated by arrow) negative for FSH. × 400.

cribed by Pretl in 1944 and further studied by Feyrter in 1951. The importance of the prostate gland as an endocrine organ is supported by the recent identification of several peptides in this gland, including endorphins (Tsong *et al* 1982), vasopressin and oxytocin (Adashi & Hsueh, 1981), relaxin (Cameron *et al*., 1982), somatostatin (Di Sant Agnese & de Mesy-Jensen, 1984), inhibin (Sathe *et al*., 1987; Vanage *et al*., 1989) and insulin (Stahler *et al*., 1988). Contrary to earlier concepts, it now appears that regulatory peptides are widely distributed and are involved in various functions that are beyond those classically recognised for these peptides. In correlation with this concept, our present study demonstrates immunocytochemical staining for FSH or FSH-like peptide in epithelial cells of human prostate, whether normal benign or malignant. The specificity of staining for immunoreactive FSH is established by positive controls (pituitary and testis) and negative controls (oesophagus, urinary bladder, rectum and epididymis). Our findings are in agreement with earlier observation made by Harper and Griffith (1982), who reported positive immunocytochemical localisation of FSH in the prostatic specimens from 11 BPH patients. In the present study, weakly positive staining of stromal cells was observed in few specimens from benign and malignant prostates. In our other studies, we have observed stromal cells showing strong positive staining for hLH and rather faint in epithelial cells (unpublished data). In the light of the above observation, whether the weak immunochemical staining observed for FSH in stromal cells of some samples is due to a common alpha-subunit of FSH and LH remains to be determined.

Epithelial cells from normal prostate showed a positive reaction, indicating the presence of immunoreactive FSH during the prepubertal stage. The maximum intensity was observed in cases of BPH where the staining was granular and coarse. The blob-like secretion of immunoreactive FSH often seen in BPH sections provides an explanation for the presence of FSH in human semen reported earlier (Biswas *et al.*, 1978; Fossati *et al.*,1979). In both moderately and poorly differentiated carcinomas, the positive reaction was focal and of varied intensity within the same tumour. As compared to other prostatic antigens, i.e. prostatic acid phosphatase and prostate specific antigen, we have previously shown that prostatic inhibin peptide appears to be a vital product as it persists in epithelial cells of poorly differentiated prostatic carcinoma when these cells have become negative for the above mentioned antigens (Sheth *et al.*, 1988). In confirmation of the relationship of inhibin and FSH, we find positive cells in all poorly differentiated carcinomas. The positive staining of immunoreactive FSH in metastatic lymph nodes with confirmed prostatic carcinoma is indicative of the spread of prostatic epithelial cells. Pending further studies on the metastasis of non-prostatic tumours, at the present stage we do not propose FSH as a specific marker for prostatic tumour.

The changes in the intensity as well as the pattern observed for FSH and inhibin (Doctor *et al.*, 1986; Sheth *et al.*, 1987) were similar. With this knowledge, it is tempting to speculate that in the prostate FSH plays a regulatory role in inhibin biosynthesis. This is in agreement with our earlier observation that exogenous/endogenous FSH is involved in the modulation of inhibin concentrations of rat prostate (T.R. Teni *et al.*, in preparation).

Inhibin has been well demonstrated to be involved in prostatic growth and differentiation by virtue of its effect of polyamine biosynthesis (Natraj *et al.*, 1986) and steroidogenesis (Joseph *et al.*, 1987). It is therefore plausible that FSH, either directly or by modulating inhibin levels, could be involved in the aetiopathology of the prostate. Earlier studies in animals indicated that pituitary hormones could influence prostatic growth and function, since a more marked atrophy of the rat prostate gland was observed after hypophysectomy and castration, than after castration alone (Lostroh *et al.*, 1957). However, information as to which of the pituitary hormones were involved was not available. It is likely that among pituitary hormones prolactin, GH and now FSH may be the hormones related to prostatic dysfunction and may possibly find clinical application in therapeutic decisions.

The immunocytochemical identification of FSH-like peptide in the epithelium of prostate tissue could be due either to circulating FSH bound to specific tissue receptors which then accumulates intracellularly, or to local production of FSH by the tissue. Our results, however, indicate a lack of specific FSH receptors on human BPH tissue. Furthermore, recent studies carried out by us have revealed the incorporation of radiolabelled leucine into hFSH which was immunoprecipitated by specific antisera to hFSH, thus indicating *de novo* biosynthesis of FSH by human BPH tissue in an *in vitro* system. Hence the high intensity of FSH staining in the cytoplasm of epithelial cells (particularly in BPH) observed by us in the present study is due to synthesis and not internalisation of circulatory FSH. It is therefore worthwhile to investigate the regulatory mechanism involved in the biosynthesis of FSH in human prostate. It may be mentioned that both pituitary and prostate show similar intensity of FSH staining (antiserum dilution 1:200). It is not yet known whether FSH synthesised by prostate is bioactive and physicochemically similar to pituitary FSH. Furthermore, although prostatic FSH does get secreted in semen (Biswas *et al.*, 1978; Fossati *et al.*, 1979) it is not yet elucidated whether or not it enters the circulation. Recently Yoon *et al.* (1987) have demonstrated the immunocytochemical localisation of FSH in human testis. This report supports our findings that FSH can be synthesised by non-pituitary tissues.

In view of the well established growth regulatory role of FSH in gonads and its role in modulating inhibin biosynthesis in human prostate glands (Vanage *et al.*, 1989) it would be of interest to evaluate the autocrine or paracrine roles of FSH in the aetiopathology of prostatic diseases.

The reagents used in these studies were supplied by the National Institutes of Health, US Department of Health and Human Services under agreement no. 01-051, the activities of the Indo-US Subcommission on Science and Technology and Dr R.S. Raiti of the National Institute of Diabetes and Digestive and Kidney Diseases (NIDDK) and National Hormone and Pituitary Program, University of Maryland School of Medicine, USA. The authors thank Dr Usha Joshi for supplying antibodies used for part of these studies. The award of a Research Associateship to one of the authors (S.V.G.) by the Council of Scientific and Industrial Research (CSIR), New Delhi, is acknowledged.

### References

ADASHI, E. & HSUEH, A.J.W. (1981). Direct inhibition of testicular androgen biosynthesis revealing antigonadal activity of neurohypophysial hormones. *Nature*, **293**, 650.

BISWAS, S., FERGUSON, K.M., STEDRONSKA, J., BAFFOE, G., MANISFIELD, M.D. & KOSBAB, M.H. (1978). Fructose and hormone levels in semen: Their correlations with sperm counts and motility. *Fertil. Steril.*, **30**, 200.

CAMERON, D.F., CORTON, G.L. & LARKIN, L.H. (1982). Relaxin-like antigenecity in the armadillo prostate gland. *Ann. NY Acad. Sci.*, **380**, 231.

DI SANT AGNESE, P.A. & DE MESY-JENSEN, J.L. (1984). Somatostatin and/or somatostatin-like immunoreactive endocrine-paracrine cells in human prostate gland. *Arch. Pathol. Lab. Med.*, **108**, 693.

DOCTOR, V.M., SHETH, A.R., SIMHA, M.M., ARBATTI, N.J., ZAVERI, J.P. & SHETH, N.A. (1986). Studies on immunocytochemical localization of inhibin-like material in human prostatic tissue: comparison of its distribution in normal, benign and malignant prostates. *Br. J. Cancer*, **53**, 547.

FEYRTER, F. (1951). Uber des urogenitale Helle-zelle-system des menschen. *Mikrosk. Anat. Forsch.*, **57**, 324.

FOSSATI, P., ASFOUR, M, BLACKER, C., BOUTEMY, J.J. & HERMAND, E. (1979). Serum and seminal gonadotropins in normal and infertile men. Correlations with sperm count, prolactinemia and seminal prolactin. *Arch. Androl.*, **2**, 247.

HARPER, M.E. & GRIFFITH, K. (1982). Protein hormones and prostate cancer. In *Prostate Cancer*, Jacobi, G.H. & Hohensellner, R. (eds) p. 409. Williamson, & Wilkins: Baltimore.

JOSEPH, R., MAITRA, A., MOODBIDRI, S.B. & SHETH, A.R. (1987). Effect of inhibin on testosterone metabolism by rat ventral prostate *in vitro*. *Arch. Androl.*, **18**, 205.

LOSTROH, A.J. & LI, C.H. (1957). Stimulation of the sex accessories of hypophysectomized male rats by non-gonadotropic hormones of the pituitary gland. *Acta Endocrinol.*, **25**, 1.

NATRAJ, U., VANAGE, G.R., DIDOLKAR, A.K. MOODBIDRI, S.B. & SHETH, A.R. (1986). Stimulation of ornithine decarboxylase in rat prostate with inhibin. *Int. J. Androl.*, **9**, 218.

PHADKE, M.A., VANAGE, G.R. & SHETH, A.R. (1987). Circulating levels of inhibin, prolactin, TSH, LH and FSH in benign prostatic hypertrophy before and after tumour resection. *Prostate*, **10**, 115.

PRETL, K. (1944). Zur Frage der endokrinie de menschleichen vorsteherdruse vrichows. *Arch. Pathol. Anat.*, **312**, 392.

REICHERT, L.E. Jr & BHALLA, V.K. (1974). Development of a radioligand-receptor assay for human follicle stimulating hormone. *Endocrinology*, **94**, 483.

REICHERT, L.E. & ABOU-ISSA, H. (1977). Studies on low molecular weight testicular factor which inhibits binding of FSH to receptor. *Biol. Reprod.*, **17**, 614.

SATHE, V.S., SHETH, N.A., PHADKE, M.A., SHETH, A.R. & ZAVERI, J.P. (1987). Biosynthesis and localization of inhibin in human prostate. *Prostate*, **10**, 33.

SHETH, N.A., DOCTOR, V.M., SAMPAT, M.B., GARDE, S.V., ARBATTI, N.J. & SHETH, A.R. (1987). Inhibin-like material. An immunohistochemical marker for prostatic origin of metastases. *Cancer Lett.*, **36**, 93.

SHETH, A.R., TENI, T.R. & SHETH, N.A. (1988). Human prostatic inhibin. In *Progress in Endocrinology*, Imura, H., Kazuo, S. & Sho, Y. (eds) p. 301. Elsevier Science Publishers: Amsterdam.

STAHLER, M.S., PANSKY, B. & BUDD, G.C. (1988). Immunocytochemical demonstration of insulin or insulin-like immunoreactivity in the rat prostate gland. *Prostate*, **13**, 189.

VANAGE, G.R., PHADKE, M.A., BANDIVDEKAR, A.H. & SHETH, A.R. (1989). Hormonal modulation of *in vitro* biosynthesis of inhibin like peptide from human prostate. *Andrologia* (in the press).

VAZE, A.Y., THAKUR, A.N. & SHETH, A.R. (1979). Development of a radioimmunoassay for human seminal plasma inhibin. *J. Reprod. Fertil. Suppl.*, **26**, 135.

TSONG, S.D., PHILLIPS, D., HALMI, N., LIOTTA, A.S., MARGIORIS, A. & BARDIN, C.W. (1982). ACTH and beta-endorphin related peptides are present in multiple sites in the reproductive tract of the male rat. *Endocrinology*, **110**, 2204.

YOON, D.J., MIRCEA, G., ROGER, S., BRUCE, S., CHARLES, A.S. & RAPHAEL, D. (1987). Immunocytochemical localization of hFSH as an index of Sertoli cell function in the human testis. *Acta Endocrinol.*, **116**, 333.